# Motivation Scale for Using Social Network Sites: Comparative Study between Facebook, Instagram, Twitter, Snapchat and LinkedIn

**DOI:** 10.5334/pb.1161

**Published:** 2023-04-11

**Authors:** Alexandra Masciantonio, David Bourguignon

**Affiliations:** 1 Universitéde Lorraine, EA 7312 PErSEUs, Metz, FR; 2 Maastricht University, Studio Europa Maastricht, Maastricht, NL

**Keywords:** motivation, Facebook, Instagram, Twitter, Snapchat, LinkedIn.

## Abstract

The increasing number of Social Network Sites (SNSs) and their changing nature raise the question of why people use them. This research has a twofold objective: first, to develop a motivation scale for using SNSs; second, to compare the motivational SNSs profile of Facebook, Instagram, Twitter, Snapchat and LinkedIn. Two studies on 364 university students, using exploratory and confirmatory factor analyses, established six motivations: entertainment, social interaction, seeking information, instrumental use, self-documentation and self-enhancement. Regressions then examined the association between motivations for using SNSs, social influence measures (descriptive and injunctive norms), and frequency of use of Facebook, Instagram, Twitter, Snapchat, and LinkedIn. The results showed that social norms complement the motivations to use SNSs. Twitter use was associated with an information-seeking SNSs motivational profile. LinkedIn, Facebook, and Instagram were associated with self-documentation on SNSs. Snapchat was rather associated with instrumental motivations on SNSs. However, while all SNSs were associated with descriptive norms, only Facebook and LinkedIn were associated with injunctive norms (i.e., peer pressure). The results are discussed by applying a cross-media perspective to new motives behind SNSs use.

## Introduction

With over 3 billion users in the world (*Global Social Media Research Summary 2019*, 2019), the popularity of Social Network Sites (SNSs) continues to expand every year. A consensual definition describes them as

networked communication platform in which participants 1) have uniquely identifiable profiles that consist of user-supplied content, content provided by other users, and/or system-level data; 2) can publicly articulate connections that can be viewed and traversed by others; and 3) can consume, produce, and/or interact with streams of user-generated content provided by their connections on the site. (Ellison & boyd, 2013, p. 157)

This increasing popularity raises numerous issues. For example, some studies have shown that using SNSs may improve well-being (Apaolaza et al., 2013; Valenzuela et al., 2009), while others have demonstrated just the opposite (Lin et al., 2016; Sagioglou & Greitemeyer, 2014). At the same time, Rae and Lonborg (2015) showed that taking into account Facebook users’ motivations provides an explanation for the inconsistent relation between quantity of Facebook use and well-being. Doing so, answering to the question “Why people use SNSs?” appears to be a major topic to integrate some inconsistencies in the literature. The purpose of the present research is therefore to better identify the motivations behind SNSs utilization. The second objective is to compare the motivational SNSs profile of Facebook, Instagram, Twitter, Snapchat, and LinkedIn; while exploring the extent to which social norms add up to these profiles.

Motivation can be defined as “the active direction of behavior toward certain preferred categories of situations or objects” (Nuttin, 1996, p. 14). It is possible to differentiate the content of motivations – the objective to achieve – from motivational mechanisms – the cognitive processes behind this behavior. Many theories in psychology have focused on human motivation (Fenouillet, 2012). For example, Vignoles et al. (2006,2008) consider that there are six needs necessary for the construction of identity: self-esteem, continuity, distinctiveness, meaning, efficacy and belonging. Regarding the literature on SNSs, the most prominent approach is *Uses-and-Gratifications Theory* (UGT, Katz et al., 1973). According to this perspective, users are aware of their motives and choose in consequence the media that gratifies the most their needs. UGT focuses therefore on the content of the motivations to use SNSs.

Although several studies investigated SNSs use in general (Barker, 2009; Pelling & White, 2009), SNSs exhibit differences (Bossetta, 2018). Consequently, researchers tend increasingly to focus on motives for using one specific SNS. Investigations have primary explored Facebook and Twitter (Nkwe, 2018). For instance, Cheung et al. (2011) showed that Facebook is used to maintain interpersonal interconnectivity, to provide social enhancement and to be entertained. Nadkarni and Hofmann (2012) reviewed the literature on the factors contributing to Facebook use and proposed a model based on two independent primary needs: the need to belong and the need for self-presentation. In other words, Facebook allows people to find social support and to display their idealized selves. In contrast, Johnson and Yang (2009) revealed that Twitter is mainly used to satisfy informational motives rather than social motives. Twitter users don’t search primarily to have fun or to keep in touch with their relatives, they seek to get information, to learn interesting things and to share information. Similarly, opinion leaders on Twitter are motivated by the platform’s possibilities such as public expression, mobilization and seeking information (Park, 2013).

However, there is a multiplication of SNSs over the last two decades and few researchers have explored motives for using other prominent SNSs, like Instagram, Snapchat or LinkedIn. Sheldon and Bryant (2016) found that Instagram use is related to surveillance about others, documentation, coolness and creativity. M. Kim and Cha (2017) showed that motives to use LinkedIn are professional advancement and self-presentation. The motives for using Snapchat are procrastination, keeping in touch with relatives and seeing what people are up to (Utz et al., 2015). Alhabash and Ma (2017) are one of the few to compare several SNSs (Facebook, Twitter, Instagram, and Snapchat): for all platforms, they found that entertainment was the main motivation. They also highlighted differences across all platforms, for instance self-expression was only associated with the use of Instagram. It must be noted that the nature of SNSs is also changing over time. For example, Facebook added an online sell platform in 2017 and Twitter has been in the spotlight with several collective movements (Saltiel, 2018; Theocharis et al., 2015).

Besides the changing nature and the multiplication of SNSs, one issue in the literature is the lack of a unified measure: some researchers argue for two motivations (Johnson & Yang, 2009), others for three (Rae & Lonborg, 2015), four (M. Kim & Cha, 2017; Sheldon & Bryant, 2016), five (Al-Menayes, 2015; Cheung et al., 2011; Gao & Feng, 2016; M. Kim & Cha, 2017; Y. Kim et al., 2011), or seven (Alhabash & McAlister, 2015). In most cases, authors have used validation techniques to develop their motivations’ scale – principal components analysis, eigenvalues greater than one, or orthogonal rotation – but these statistical methods, which lead to retain too many factors, are no longer recommended (for a discussion, see Carpenter, 2018).

Finally, a criticism often leveled against the UGT approach is that users may not be fully aware of why they choose to use SNSs. It is possible that other factors influence their choice (Pelling & White, 2009). A complementary approach, though under-explored, includes social norms (Montag et al., 2021). Indeed, SNSs are eminently social: even the personal profile is co-constructed (Ellison & boyd, 2013). Like motivations, social norms have been the subject of numerous theories in psychology (Girandola & Fointiat, 2016). One theory, in particular, seems to be of interest in the context of SNSs: Focus Theory of Normative Conduct (Cialdini et al., 1991). According to the latter, there are two types of norms: injunctive and descriptive. The first derives from “the perception of what most people approve or disapprove” (Cialdini et al., 1991, p. 203) and corresponds to social pressure to use SNSs. The second derives from “what other people do” and corresponds to the perception that individuals have of other users on SNSs (Cialdini & Trost, 1998, p. 155). This model has already shown its value in understanding self-expression on Facebook, Instagram, Twitter, and WhatsApp (Waterloo et al., 2018). This raises the question of the extent to which social norms complement users’ motivations.

### The present research

Taken together, the changing nature of SNSs along with their proliferation require to explore why people use SNSs. The purpose of this research was therefore to develop a new motivation scale for using SNSs. In addition, a comparative study between Facebook, Instagram, Twitter, Snapchat and LinkedIn has been also carried out. However, to be consistent with the research in the field and for reasons of sampling convenience, the scale was validated in two university samples. Although not all university students are necessarily young, this choice certainly limits the scope of this research.

The choice of these five SNSs reflects a need to extend the literature which generally focuses on one single platform (Bank & Lutz, 2017), but also a consideration of their specific features. Indeed, Instagram and Snapchat are based on image, but the first is unidirectional (i.e., everyone can access the user’s profile even without prior approval) while the second is dyadic (i.e., the user needs to approve a person before they can access to his or her profile). Twitter is based on text and is unidirectional. Both Facebook and LinkedIn are based simultaneously on text and image, but Facebook is dyadic and LinkedIn is unidirectional (Pittman & Reich, 2016). Facebook, Instagram, Twitter, and Snapchat are also recognized as general SNSs (Alhabash & Ma, 2017), while LinkedIn is considered as a professional SNS. Doing so, Facebook, Instagram, Twitter, Snapchat, and LinkedIn capture quite well the actual landscape of SNSs.

Based on a preliminary study to generate items, study 1 extracted factors of motivation by conducting an exploratory/common factor analysis. In study 2, confirmatory factor analysis was carried out to verify the structure of the motivation scale for using SNSs. In order to verify how social norms complement these new motivations, regressions were then conducted on the frequency of use of Facebook, Instagram, Twitter, Snapchat, and LinkedIn. The data and the codebooks of the two studies are openly available in Open Science Framework (see “Open Science section”).

## Study 1

### Method

#### Participants

257 first-year French psychology students completed a self-administered questionnaire during a lecture course. Participation was voluntary and all participants signed an informed consent form. Participants were 41 men, 210 women and 4 persons with another gender identity (mean age = 19.02 years, *SD* = 2.97, range = 17 to 41). 2 respondents didn’t complete sociodemographic questions. The research took place in France, so all items were in French. Throughout the manuscript, items were therefore translated in English.

#### Procedure and materials

##### Selection of a sample of items

A preliminary study was conducted to identify potential items of the scale. First, drawing on the literature (Alhabash & McAlister, 2015; Cheung et al., 2011; Johnson & Yang, 2009; Y. Kim et al., 2011; Sheldon & Bryant, 2016; Shi et al., 2013), 33 items were chosen based on their relevance and to avoid redundancy. Second, to obtain new motives behind SNSs use, a qualitative study was conducted by posting the URL to an anonymous questionnaire on Facebook. The sample comprised 79 respondents, 16 men and 63 women (mean age = 33.89 years, *SD* = 10.73, range = 20 to 60), 83.54% were professionally active and others were students or unemployed. Participants rated on a five-point scale (1 = strongly disagree, 5 = strongly agree) how much the 33 items previously selected in the literature correspond to their reasons for using SNSs. Then, an open-ended question asked them if one or more of their motives for using SNSs were missing. All open-ended answers were taken into account, except when they were redundant with other motivations. By this qualitative study, 6 additional items were identified. It should be noted that the average age of participants in our qualitative study is higher than that of the samples in Studies 1 and 2. However, this study was purely exploratory in nature and aimed to identify items that may be missing rather than to test theories. The 39 potential items generated are presented in Table 1.

**Table 1:** Sources used to generate initial pool of items.


ITEMS	SOURCES

To meet new people	Jonhson & Yang (2009)

To participate in discussions	

To express myself freely	

To communicate more easily

To communicate with many people at the same time

To get information	Cheung, Chiu, & Lee (2011)

To negotiate or bargain	

To be entertained	

To impress	

To let others know I care about their feelings	Y. Kim, Sohn, & Choi (2011)

To talk out my problems and get advice	

To get new ideas	

To learn about unknown things	

To relax	

To pass time	

To forget about work or other things	

To get what I want for less effort	

To keep in touch with my family	Shi, Yue, & He (2013)

To keep in touch with my old friends	

Because I think it might be helpful for my work	

To record what I do in life	Alhabash & McAlister (2015)

To record what I have learned	

To record where I have been	

To share information	

To show my personality	

To tell others about myself	

Because I like that I can post things I want to say immediately	

To remember special events	Sheldon & Bryant (2016)

To see what other people share	

To find people with whom I have common interests	

To create art	

To become popular	

To self-promote	

To find old friends	Qualitative study

To find love and/or flirt	

To follow the news	

To purchase online	

To sell online	

To build a professional network	


##### Test of the sample of items

Participants responded on a five-point scale (1 = strongly disagree, 5 = strongly agree) how much each of the 39 items selected by the preliminary study corresponds to their reasons for using SNSs. They were asked for SNSs in general, no example of SNS was given. Sociodemographic questions were also added with respect to age and gender.

### Results and brief discussion

In line with the recommendations of Carpenter (2018), an exploratory/common factor analysis with a principal axis method and a Promax rotation was conducted on JASP (JASP Team, 2020). Since the eigenvalue-greater-than-one rule is no longer recommended (O’connor, 2000), the number of components to retain was determined by parallel analysis. The cutoff criterion was a minimal significant loading above .40 (Carpenter, 2018), nine items did not meet this criterion and were dropped: “To keep in touch with my family”, “To keep in touch with my old friends”, “To find old friends”, “To see what other people share”, “To find love and/or flirt”, “To share information”, “To get what I want for less effort”, “To communicate more easily”, and “Because I like that I can post things I want to say immediately”. No items saturated on several factors with a factor loading above .40.

Using the same approach, the 30 items remaining were analyzed. All loaded above .40 on one unique factor. The Kaiser-Meyer-Olkin values were between .72 to .90 and the Bartlett’s test of sphericity was significant (*p* < .001). Six factors were extracted, accounting for 52.7% of the variance. These factors were labeled: *social interaction, entertainment, self-documentation, instrumental use, seeking information*, and *self-enhancement*. To estimate the internal consistency, the McDonald’s ω was preferred over the Cronbach’s α (Béland et al., 2017). All McDonald’s ω were above .80. Table 2 summaries results of this analysis. In addition, Table 3 shows the factor correlations.

**Table 2:** Summary of exploratory/common factor analysis results for the motivation scale for using SNSs.


	SOCIAL INTERACTION	ENTERTAINMENT	INSTRUMENTAL USE	SELF-DOCUMENTATION	INFORMATION SEEKING	SELF-ENHANCEMENT

To find people with whom I have common interests.	**0.756**	–0.034	–0.025	–0.131	0.042	–0.045

To talk out my problems and get advice.	**0.655**	–0.020	–0.046	0.062	–0.097	–0.093

To tell others about myself.	**0.601**	0.003	–0.060	0.023	–0.040	0.270

To express myself freely.	**0.593**	0.118	0.009	–0.019	0.094	0.039

To show my personality.	**0.564**	0.035	–0.093	0.032	0.087	0.186

To let others know I care about their feelings.	**0.548**	0.027	–0.109	0.108	0.092	–0.076

To participate in discussions.	**0.543**	0.087	0.326	–0.121	–0.111	–0.110

To meet new people.	**0.528**	0.041	0.006	–0.024	0.066	–0.029

To create art.	**0.525**	–0.167	0.021	0.063	–0.076	0.096

To communicate with many people at the same time.	**0.447**	0.284	0.070	–0.031	0.043	–0.095

To be entertained.	0.044	**0.809**	–0.003	0.023	0.068	0.075

To relax.	–0.036	**0.737**	–0.021	–0.019	0.033	0.063

To forget about work or other things.	0.032	**0.738**	0.029	0.086	–0.152	0.028

To pass time.	0.026	**0.797**	–0.086	0.015	–0.011	0.001

Because I think it might be helpful for my work.	0.249	–0.034	**0.514**	0.060	–0.041	–0.052

To purchase online.	–0.192	0.137	**0.632**	0.069	0.167	0.004

To sell online.	–0.167	0.036	**0.873**	0.025	0.083	0.005

To negotiate or bargain.	–0.017	–0.054	**0.687**	–0.047	0.031	0.036

To build a professional network.	0.158	–0.218	**0.594**	0.037	–0.112	0.134

To record where I have been.	–0.106	0.090	0.076	**0.826**	–0.092	0.007

To record what I do in life.	0.068	0.018	–0.089	**0.809**	–0.035	–0.033

To remember special events.	–0.013	0.102	0.110	**0.742**	–0.085	–0.010

To record what I have learned.	0.083	–0.145	–0.013	**0.617**	0.254	–0.046

To get new ideas.	0.071	–0.047	0.017	0.101	**0.630**	0.072

To follow the news.	–0.096	0.011	0.094	–0.079	**0.662**	–0.047

To learn about unknown things.	–0.005	–0.076	–0.057	0.047	**0.948**	0.061

To get information.	0.142	0.029	0.065	–0.094	**0.575**	–0.053

To self-promote.	–0.115	0.075	0.039	–0.019	–0.004	**0.952**

To become popular.	–0.060	0.125	0.058	0.005	0.017	**0.790**

To impress.	0.166	–0.047	–0.001	–0.066	0.007	**0.700**

% of variance	12.3	8.9	8.3	8.0	7.7	7.5

McDonald’s ω	.85	.85	.81	.85	.81	.86


*Note*: Applied rotation method is promax. Factor loadings over .40 appear in bold.

**Table 3:** Factor correlations for the motivation scale for using SNSs.


	SOCIAL INTERACTION	ENTERTAINMENT	INSTRUMENTAL USE	SELF-DOCUMENTATION	INFORMATION SEEKING	SELF-ENHANCEMENT

**Social interaction**	1.000	0.362	0.443	0.418	0.372	0.373

**Entertainment**	0.362	1.000	0.142	0.221	0.400	0.014

**Self-documentation**	0.443	0.142	1.000	0.262	0.339	0.213

**Instrumental use**	0.418	0.221	0.262	1.000	0.272	0.185

**Information seeking**	0.372	0.400	0.339	0.272	1.000	-0.078

**Self-enhancement**	0.373	0.014	0.213	0.185	–0.078	1.000


This first study allowed us to identify six motivations for using SNSs. However, the development of a measurement scale requires the exploratory factor analysis to be followed by confirmatory factor analysis (Carpenter, 2018). In addition, the literature shows that women and men use SNSs differently (Hanna et al., 2017; Twenge & Martin, 2020), but our sample is predominantly of women. Therefore, the second study aims to validate the scale through confirmatory factor analysis among a balanced sample. Moreover, one can wonder to what extent other factors – of which individuals are not aware – can impact their reasons for using SNSs. Specifically, the literature highlights that social influence can be an important factor in whether to use an SNS (Montag et al., 2021). Therefore, the second study also aims to explain the frequency of use of Facebook, Instagram, Twitter, Snapchat, and LinkedIn through the six motivations for using SNSs and social norms.

## Study 2

### Method

#### Participants

The second sample comprised 107 first-year French information and communication students. They completed a self-administered questionnaire during a lecture course. Participation was voluntary and all participants signed an informed consent form. Participants were 56 men, 45 women and 2 persons with another gender identity (mean age = 18.85 years, *SD* = 1.61, range = 17 to 26). 4 respondents didn’t complete sociodemographic questions. 85.05% participants held a Facebook account, 88.79% held an Instagram account, 78.10% held a Twitter account, 93.46% held a Snapchat account and finally, 23.36% held a LinkedIn account. Regarding the SNSs frequency of use, LinkedIn (mean = 1.25, *SD* = .63) was the least used, followed by Facebook (mean = 2.58, *SD* = 1.33). Snapchat (mean = 4.10, *SD* = 1.32) and Instagram (mean = 4.02, *SD* = 1.39) were the most frequently used, followed by Twitter (mean = 3.48, *SD* = 1.74). This is consistent with studies of young adults (Boczkowski et al., 2018).

#### Procedure and materials

*Motivation to use SNSs*. Participants were asked to indicate on a five-point scale the extent to which the items identified in the first study correspond to their motivations for using SNSs (1 = Strongly disagree; 5 = Strongly agree).

*SNS use*. The measure was adapted from Nick et al. (2018). Participants were asked a single question about how frequently they use Facebook, Instagram, Twitter, Snapchat and LinkedIn on a five-point scale (1 = never, 1 = rarely, 3 = sometimes, 4 = pretty often, 5 = a lot).

*Social influence for using SNS*. Two measures of social influence were assessed: injunctive norm and descriptive norm (Cialdini & Trost, 1998). The measure of injunctive norms was adapted from Posey et al. (2010) with three items per SNSs (e.g., “People who are important to me think that I should use Facebook”). Responses were made on five-point scale (1 = strongly disagree, 5 = strongly agree). McDonald’s ω computed a value of .85 for Facebook, .86 for Instagram, .88 for Twitter, .91 for Snapchat and .92 for LinkedIn. The measure of descriptive norm was defined as the perceived frequency of SNSs use for family and for friends. Respondents were asked how frequently their family use Facebook, Instagram, Twitter, Snapchat and LinkedIn on a five-point scale (1 = never, 1 = rarely, 3 = sometimes, 4 = pretty often, 5 = a lot). They were asked the same questions for their friends.

### Results and brief discussion

#### Motivations to use SNSs

To establish how well the structure of the motivation scale for using SNSs fits to the observed data, confirmatory factor analysis was conducted using Lavaan (Rosseel, 2012) and JASP (JASP Team, 2020). The DWLS (Diagonally Weighted Least Squares) estimator, which is adapted for small sample with data violating normality, was chosen (Gana & Broc, 2018).

All standardized item loadings exceeded .3 and differed reliably from zero (*p* < .001), expect one item of the social interaction’s motivation (.29; *p* < .001). Five fit indices were also used, all suggested a very good fit to the data: χ^2^ = 290.945, *p* = 1.000; SRMR = .085; RMSEA = .000; CFI = 1.000; TLI = 1.051. McDonald’s ω were .81 for *entertainment*, .83 for *social interaction*, .80 for *seeking information*, .81 for *instrumental use*, .83 for *self-documentation*, .92 for *self-enhancement*.

#### Comparison of five SNSs

The second aim of the research was to compare the motivational SNSs profile of Facebook, Instagram, Twitter, Snapchat, and LinkedIn; while exploring the extent to which social norms add up to these profiles. Using JASP (JASP Team, 2020), we began by regressing the six motivations on the frequency of use of each SNS. This provided a SNSs motivation profile associated with each SNS. Then, we redid the same analyzes but added social norms. Each time, we checked for socio-demographic variables (gender,^1^ age). Backward regressions were chosen because they are appropriate for exploratory model (Field, 2013).

##### Frequency of Facebook use

Table 4 shows the SNSs motivational profile associated with Facebook is characterized by using SNSs for social interaction. In addition, men are using more Facebook than persons with other gender identity.

**Table 4:** Results at the regression’s final stage for frequency of Facebook use.


	*B*	*SE B*	*β*

Constant	1.827	0.441	

**Social Interaction**	0.277	0.147	0.183*

**Men vs. Other** (dummy variable)	–1.653	0.924	–0.174*


*Note*: *R^2^* = .063 for Step 8. * *p* < .1 ** *p* ⩽ .05 *** *p* ⩽ .001.

However, when adding social norms variables, the results changed. Table 5 shows that 47% of the variance of frequency of Facebook use was explained by six variables: SNSs self-documentation, SNSs injunctive norm, family descriptive norm, friend descriptive norm, men *vs*. women and men *vs*. other gender identity.

**Table 5:** Results at the regression’s final stage for frequency of Facebook use with social norms.


	*B*	*SE B*	*β*

Constant	–0.789	0.492	

**Self-documentation**	0.200	0.099	0.154**

**Men vs. Women** (dummy variable)	–0.359	0.203	–0.138*

**Men vs. Other** (dummy variable)	–1.566	0.722	–0.168**

**Friends descriptive norm**	0.472	0.099	0.384***

**Family descriptive norm**	0.308	0.083	0.300***

**Injunctive norm**	0.288	0.125	0.185**


*Note*: *R^2^* = .47 for Step 7. * *p* < .1 ** *p* ⩽ .05 *** *p* ⩽ .001.

##### Frequency of Instagram use

Concerning Instagram, the frequency of use is associated with a SNSs motivational profile characterized by using SNSs for self-documentation, entertainment and instrumental use (Table 6).

**Table 6:** Results at the regression’s final stage for frequency of Instagram use.


	*B*	*SE B*	*β*

Constant	0.410	0.706	

**Self-documentation**	0.296	0.139	0.210**

**Entertainement**	0.418	0.173	0.241**

**Instrumental use**	0.296	0.122	0.218**


*Note*: *R^2^* = .23 for Step 7. * *p* < .1 ** *p* ⩽ .05 *** *p* ⩽ .001.

Contrary to Facebook, the motivations for using SNSs are the same when adding social norms in the model. Table 7 shows that 40% of the variance of frequency of Instagram use was explained by five variables: entertainment, self-documentation, instrumental use, friend descriptive norm and family descriptive norm.

**Table 7:** Results at the regression’s final stage for frequency of Instagram use with social norms.


	*B*	*SE B*	*β*

Constant	–2.482	0.870	

**Self-documentation**	0.240	0.127	0.171*

**Entertainement**	0.436	0.155	0.254**

**Instrumental use**	0.316	0.109	0.237**

**Friends descriptive norm**	0.574	0.141	0.337***

**Family descriptive norm**	0.185	0.103	0.151*


*Note*: *R^2^* = .40 for Step 8. * *p* < .1 ** *p* ⩽ .05 *** *p* ⩽ .001.

##### Frequency of Twitter use

Twitter frequency of use is associated with going on SNSs to seek information. Moreover, the younger the users, the more they tend to use Twitter (Table 8).

**Table 8:** Results at the regression’s final stage for frequency of Twitter use.


	*B*	*SE B*	*β*

Constant	4.447	1.964	

**Seeking information**	0.784	0.216	0.348***

**Age**	–0.221	0.103	–0.207**


*Note*: *R^2^* = .134 for Step 10. * *p* < .1 ** *p* ⩽ .05 *** *p* ⩽ .001.

Again, SNSs motivations profile for Twitter is similar when considering social norms in the model. Table 9 shows that 36% of the variance of frequency of Twitter use was explained by three variables: seeking information, friend descriptive norm and age.

**Table 9:** Results at the regression’s final stage for frequency of Twitter use with social norms.


	*B*	*SE B*	*β*

Constant	1.143	1.804	

**Seeking information**	0.757	0.188	0.336***

**Age**	–0.204	0.089	–0.190**

**Friends descriptive norm**	0.734	0.127	0.473***


*Note*: *R^2^* = .36 for Step 10. * *p* < .1 ** *p* ⩽ .05 *** *p* ⩽ .001.

##### Frequency of Snapchat use

As for Twitter, Snapchat users seem to be younger. In addition, the use of the platform is associated with SNSs instrumental use (Table 10).

**Table 10:** Results at the regression’s final stage for frequency of Snapchat use.


	*B*	*SE B*	*β*

Constant	8.784	1.497	

**Instrumental use**	0.216	0.119	0.169*

**Age**	–0.283	0.078	–0.340***


*Note*: *R^2^* = .139 for Step 8. * *p* < .1 ** *p* ⩽ .05 *** *p* ⩽ .001.

Adding social norms to the model did not change the SNSs motivational profile. Table 11 shows that 38% of the variance of frequency of Snapchat use was explained by three variables: instrumental use, friend descriptive norm and age.

**Table 11:** Results at the regression’s final stage for frequency of Snapchat use with social norms.


	*B*	*SE B*	*β*

Constant	3.104	1.574	

**Instrumental use**	0.202	0.102	0.159**

**Age**	–0.187	0.068	–0.225**

**Friends descriptive norm**	0.857	0.139	0.504***


*Note*: *R^2^* = .38 for Step 10. * *p* < .1 ** *p* ⩽ .05 *** *p* ⩽ .001.

##### Frequency of LinkedIn use

Finally, the frequency of use of LinkedIn is associated with using SNSs for self-documentation and entertainment (Table 12). But what is interesting is that the association with entertainment is negative; in other words, the more people go on SNSs to have fun, the less likely they are to go on LinkedIn.

**Table 12:** Results at the regression’s final stage for frequency of LinkedIn use.


	*B*	*SE B*	*β*

Constant	1.465	0.318	

**Self-documentation**	0.177	0.066	0.287**

**Entertainement**	–0.197	0.081	–0.258**


*Note*: *R^2^* = .085 for Step 8. * *p* < .1 ** *p* ⩽ .05 *** *p* ⩽ .001.

Again, social norms add to the motivations for using SNSs rather than replacing them. Table 13 shows that 24% of the variance of frequency of LinkedIn use was explained by four variables: self-documentation, entertainment, injunctive norm and friend descriptive norm.

**Table 13:** Results at the regression’s final stage for frequency of LinkedIn use with social norms.


	*B*	*SE B*	*β*

Constant	0.853	0.327	

**Self-documentation**	0.161	0.061	0.262**

**Entertainement**	–0.159	0.076	–0.210**

**Friends descriptive norm**	0.160	0.074	0.200**

**Injunctive norm**	0.147	0.047	0.290**


*Note*: *R^2^* = .24 for Step 9. * *p* < .1 ** *p* ⩽ .05 *** *p* ⩽ .001.

## General discussion

This research offers certain advantages over previous work. First, exploratory and confirmatory analyses were carried out in two samples to develop the motivation scale for using SNSs. Second, this new scale was used to compare the SNSs motivational profile of five SNSs: Facebook, Instagram, Twitter, Snapchat and LinkedIn. To do so, two components of social influence were also introduced: injunctive and descriptive norms. In such ways, this research tries to respond to the rapid evolution of the SNSs landscape, where SNSs proliferate and change over the time.

The primary purpose of this research was to identify new motivations for using SNSs. Six motivations have been established: social interaction, entertainment, self-documentation, instrumental use, seeking information and self-enhancement. Consistent with the literature, findings showed that people use SNSs for entertainment, seeking information and social interaction. In addition, new motives for using SNSs were also uncovered. Self-documentation and self-enhancement are in line with the research of Sheldon and Bryant (2016). The first is a motivation for archiving daily events with the possibility to look at them at any time, like a souvenir album. The second – sometimes referred as “impression management” (Gao & Feng, 2016) – is rather a motivation for promoting oneself on SNSs, it is a form of personal branding. Instrumental use appears to be unique to this study. It refers to material resources and needed services, like purchase or help with the work. This motivation therefore embraces the professional advancement dimension of M. Kim and Cha (2017).

One surprising outcome is the absence of a motivation related to medium appeal or convenience. Indeed, previous studies have shown that individuals rely on SNSs because they are easy to use and attractive (Alhabash & Ma, 2017; Alhabash & McAlister, 2015; Al-Menayes, 2015; Y. Kim et al., 2011). Although our study does not reveal a specific factor, it is possible that convenience may explain the use of SNSs when compared to other Information and Communication Technologies, like television. Similarly, whereas previous studies identified separate motivations for seeking friends, self-expressing, providing social support or being altruism (Al-Menayes, 2015; Y. Kim et al., 2011; Sheldon & Bryant, 2016), our findings revealed one unique motivation labeled social interaction. From a statistical point of view, the use of common factor analysis (rather than principal component analysis) and parallel analysis avoided to retain too many factors (Carpenter, 2018). From a conceptual point of view, social interaction is a way of being with others. The only purpose is therefore to connect with people, whether for self-disclosing, providing social support or meeting new persons. It can be noted, however, that the motivation of social interaction contains a greater number of items than the other motivations. It would therefore be relevant to try to understand the contribution of each of these items to the dimension.

In that respect, motivations to use SNSs can be seen in parallel with human identity needs (Vignoles et al., 2006, 2008). Self-documentation corresponds to having a sense of continuity (i.e., continuity). Instrumental use allows users to become more efficient (i.e., efficacy). Seeking information may lead to the feeling that the world and oneself are meaningful (i.e., meaning). Self-enhancement is a way of seeing oneself in a positive light and distinguishing from others (i.e., self-esteem and distinctiveness). Finally, social interaction connects oneself with others (i.e., belonging). Only the entertainment seems to not match with human identity needs. This motivation leads precisely individuals to forget what is happening around them.

The second purpose of this research was to compare the SNSs motivational profile of five SNSs: Facebook, Instagram, Twitter, Snapchat and LinkedIn. Specifically, we first identified motivational SNSs profiles for each platform, and then explored the extent to which social norms add up to these profiles. Our results show that social norms did not suppress SNSs motivations; on the contrary, they complemented them. This result is particularly interesting because it highlights the importance of motivations, while demonstrating that social influence also plays a part in the reasons that lead individuals to use SNSs (Montag et al., 2021). With the exception of Facebook, the SNS motivational profiles did not change when social norms were introduced into the models. While the frequency of Facebook use was associated with the use of SNSs for social interaction, adding social norms changed this result: students Facebook’s use was associated with self-documentation, descriptive norms and injunctive norms. In other words, without social norms, we find the same results as in the literature (Nadkarni & Hofmann, 2012). However, with the inclusion of the latter, we can wonder if the use of Facebook has not changed: young users consider Facebook mainly as a recording tool and social influence prevent them from leaving the platform.

Regarding the other SNSs, participants who use Instagram reported SNSs motives to self-document and to entertain (Alhabash & Ma, 2017; Sheldon & Bryant, 2016). However, no association was found between Instagram use and social interaction, or self-enhancement. Instead, Instagram use was driven by instrumental needs. This is not surprising given current practices on the platform: users draw from celebrity profiles and “instafamous” profiles to influence their purchase behaviors (Djafarova & Rushworth, 2017). Snapchat use was also related to instrumental needs. Like Instagram, the platform is the place of a new marketing practice, called “social selling”, to engage customers through SNSs (Attia, 2017). To our knowledge, few researchers have yet explored how “social selling” affects SNSs use. A lot of studies have investigated the impacts of social media branding for the brands themselves (i.e., on purchase intentions or customer relationships, Kim & Ko, 2010), but less is known about the impacts on users’ motivations. Gao and Feng (2016) have, for instance, demonstrated that the motivation for impression management on SNS is associated with more interactions with the brand (i.e., recommend the brand to a friend, comment a publication of the brand, …). However, our findings highlight that “social selling” may directly influence users’ motivations: users may choose a SNS because they know that the platform is used for social media branding, they are looking precisely for this type of brand-related activities.

Regarding Twitter, results showed that the strongest motivation was seeking information. This is in line with the literature on Twitter utilization (Johnson & Yang, 2009; Park, 2013). Twitter and Snapchat were also the only SNSs related to age: the younger users were, the more they used these platforms. While for Snapchat, the literature points out that the platform is mainly used by young people as a kind of messaging tool, Twitter is most commonly associated with an older population (Blank & Lutz, 2017; Boczkowski et al., 2018). Nevertheless, Twitter is also particularly used by the journalistic population (Lotan et al., 2011), yet the sample in study 2 was students in information and communication sciences. The results also showed that the frequency of Facebook use is associated with gender; men tend to use Facebook more than women and people who do identify with other gender identities. This result is surprising for two reasons. On the one hand, we do not find gender-related differences for the other SNSs. On the other hand, the literature tends to show that women use Facebook, but also Instagram, more than men (Nadkarni & Hofmann, 2012; Sheldon & Bryant, 2016). It should be noted, however, that although women have long been the most active on social media, the statistics of the last few years underline that men are more present on Facebook, and are equally present on Instagram (Ben, 2023).

Finally, this research also follows the recommendation of Blank and Lutz (2017) to dedicate more attention on LinkedIn, but few university students held a LinkedIn account, and a little part of the variance was therefore explained. The platform’s utilization was positively associated with self-documentation and negatively with entertainment. As expected, LinkedIn took the form of a professional SNS where users do not search to relax, but to keep records of their professional profile.

An interesting result is that injunctive norms had a significant influence on both LinkedIn and Facebook use. In other words, individuals use these two platforms in part due to peer pressure; they believe that others expect them to do so (Cialdini & Trost, 1998). van Dijck (2013) has demonstrated that Facebook and LinkedIn are favorite spaces for performing the self. The social pressure is therefore decisive on these two platforms. The only difference is that Facebook concerns the promotion of the personal self, whereas LinkedIn concerns the promotion of the professional self. Descriptive norms had also an important bearing on each SNS use. This result has at least two meanings: first, people choose an SNSs because they can meet their relatives there, and second, they consider their friends or/and their family as good indicators of a platform’s value.

Beside these contributions, this research has some limitations. First, the scale has been validated in two university samples. Although this is conform with current studies in the field (Amichai-Hamburger & Vinitzky, 2010), it would be valuable to validate the scale in another population to extend the results. For instance, Facebook was not related to social interaction or self-enhancement, but older people may use the platform for different reasons than younger. Second, even if LinkedIn was included to have a larger picture of the current SNSs landscape, this platform is very specialized and few participants held an account. Another concern is the general measure of SNS use; individuals may use SNSs in different ways. For example, it is possible to distinguish between an active use, which aims at interacting with other users, and a passive use, which aims at consuming the content published by other users (Gerson et al., 2017). In addition, cultural context plays also a role in the motivations for using SNSs (Y. Kim et al., 2011) and the study would deserve to be replicated in a non-French population. Furthermore, self-enhancement was not related to Facebook, Instagram, Twitter, Snapchat and LinkedIn utilization. One possibility is that other SNSs, like dating sites, are more conducive to satisfy this motive. Future research should, therefore, compare more SNSs. Another important limitation is that the main objective was to develop a motivation scale for using SNSs. As such, to compare the use of Facebook, Instagram, Twitter, Snapchat, and LinkedIn, we examined whether certain motivations to use SNSs were more important when participants used one of these SNS more frequently. However, this does not allow to directly know the motivations to use these platforms. Conversely, we measured the social norms for each SNS which may lead to an imbalance in the weights of the variables; for example, regression models with social norms explain much more of the variance than those including only the motivations to use SNSs. Future research should, therefore, have the motivation scale for using SNSs completed for every SNS to be compared. Last but not least, this research has the benefit to not focus on one platform, but SNSs are still viewed as separate: it is necessary to keep in mind that people use several SNSs at the same time, hence the need for a more holistic view.

## Data Accessibility Statements

Open Data: data and codebooks are available at: https://osf.io/qs8r6/

## Footnotes

1 In this research, gender is a nominal variable with three categories (participants could choose “men”, “women” or “another gender identity”). To perform multiple regression, the gender variable was coded into two new dichotomous dummy variables. “Men” was used as the reference category because it contains the largest number of participants.
